# A randomized controlled trial of effects of open-label placebo compared to double-blind placebo and treatment-as-usual on symptoms of allergic rhinitis

**DOI:** 10.1038/s41598-023-34790-9

**Published:** 2023-05-24

**Authors:** Michael Schaefer, Kurt Zimmermann, Paul Enck

**Affiliations:** 1 Medical School Berlin, Rüdesheimer Str. 50, 14197 Berlin, Germany; 2 SymbioPharm, 35745 Herborn, Germany; 3 Department of Internal Medicine VI: Psychosomatic Medicine and Psychotherapy, University Hospital Tübingen, Osianderstraße 5, 72076 Tübingen, Germany

**Keywords:** Psychology, Immunological disorders

## Abstract

Placebo effects are known for numerous clinical symptoms. Until recently, deception of placebos was thought to be essential for placebo effects, but intriguing new studies suggest that even placebos without concealment (open-label placebos) may help patients with various clinical disorders. Most of those studies compared open-label placebo treatments with no treatment conditions (or treatment “as usual”). Given that open-label placebo studies obviously cannot be blinded, additional control studies are important to assess the efficacy of open-label placebos. The current study aimed to fil this gap by comparing open-label with conventional double-blind placebos and treatment as usual. Patients with seasonal allergic rhinitis were randomly divided in different groups. The first group received open-label placebos, the second double-blind placebos, and the third was treated as usual. After 4 weeks, results demonstrated that open-label placebos improved allergic symptoms more than treatment-as-usual and even more as double-blind placebos. In addition, we observed that allergic symptoms in general (and also the open-label placebo effects) were reduced by the Covid-19 pandemic. The results suggest that seasonal allergic symptoms may be relieved by open-label placebos. We discuss these results by addressing possible different mechanisms of open-label and conventionally concealed placebo treatments.

## Introduction

At least since the famous article about the “powerful placebo” by Beecher^1^ it has been scientifically acknowledged that placebo pills or interventions can have beneficial effects. Consequently, placebos may not only represent control conditions in randomized controlled studies in pharmacological research but also be considered with respect to therapeutic goals. Today, a significant impact of placebos have been shown in a wide variety of conditions and symptoms^2^.

Unfortunately, deception of placebos is believed to be the crucial element of a placebo effect, resulting in severe ethical problems (e.g., undermining of consent and trust between patient and healthcare provider) when trying to administer placebos. Until very recently the idea to give placebos without deception would have been considered ridiculous, but intriguing new evidence demonstrates that open-label placebos (OLPs) may help patients with various clinical disorders^3^ (and even individuals with nonclinical symptoms^4^). Thus, effects of OLPs have been shown for several different conditions, including, for example, irritable bowel syndrome, depression, pain, anxiety, and emotional distress^5–8^. Furthermore, it has been reported that OLPs may reduce symptoms in allergic rhinitis, too^9–12^.

However, most of those OLP studies represent only two-arm studies, usually the OLP and a treatment-as-usual (TAU) condition. This is problematic because of a possible different handling of the groups. Although the formal patient-experimenter interaction may be identical for both groups, the experimenter might unconsciously be more focused on participants of the OLP group and thereby providing more support to them. Given that OLP trials obviously cannot be blinded (participants know the hypothesis) and most studies report self-report data, response bias might be a consequence of these subliminal interactions. Blinded experimenters and additional control conditions such as conventional placebos would help to address this problem. This could also help to answer the question about the efficacy of an OLP treatment. Thus, reported effect sizes of OLP interventions are usually based on comparisons with TAU conditions, but it is not known whether the effect sizes of OLP are similar or lower or higher than conventional double-blind placebos. Both assumptions may be justified since the mechanisms of OLP might be different from conventional placebos. It is also important to assess the potential impact of OLPs because of possible practical implications.

The present study intended to address this gap. We aimed to test the efficacy of an OLP approach relative to a double-blind placebo, and TAU. Thus, we conducted a randomized-controlled trial on the effects of OLPs on symptoms of seasonal allergic rhinitis.

Allergic rhinitis affects up to 20% of the population in the developed world and is associated with a high economic burden^13^. It can be defined as a condition caused by a hypersensitivity of the immune system, driven by an Immunoglobulin E (IgE)-mediated reaction in the upper respiratory system. Symptoms of seasonal allergic include, for example, sneezing, rhinorrhea, or itching eyes. Although effective treatments such as immunotherapy or treatments for symptomatic relief are known^14^, they often do not completely resolve symptoms. Furthermore, they are expensive, and related to side effects^15^. Given that the symptoms of allergic rhinitis vary depending on seasonal changes with high or low allergic load in the environment, waxing and waning are characteristic for allergic symptoms. Consequently, placebo effects of symptomatic therapies are well-known^16–18^.

The current study included 89 patients with seasonal allergic symptoms. In a 4 weeks trial we tested whether an OLP treatment is superior to TAU and compare its efficacy with a conventional double blind placebo condition. The results should help us to assess the impact of an OLP treatment.

## Materials and methods

### Participants

**Figure 1:**
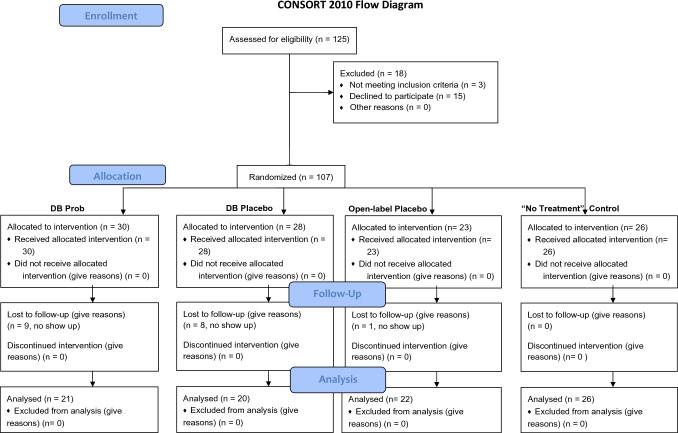
Patient flow diagram. Results for the drug part (DB Prob) of the study will be reported in a separate publication.

From springtime 2019 to springtime 2021 we screened 125 patients for eligibility. 36 patients were excluded because of not meeting the inclusion criteria and other reasons (e.g., no show up for the second measurement, see flowchart, Fig. 1). Table 1 shows demographic details of all participants. For recruitment we used flyers, social media, and the local universities. The study was stopped in spring 2020 due to the Covid-19 pandemic and continued in spring 2021. 25 of the 89 included patients participated before the pandemic.

**Table 1:** Demographics and baseline characteristics (mean ± standard deviations).

Characteristic	Open-label placebo	Double-blind placebo	Treatment-as-usual
N	22	20	26
Age (in years)	28.05 ± 10.97	26.35 ± 11.37	25.54 ± 6.76
Females/males	10/12	15/5	13/13
Duration of allergy (years)	18.00 ± 11.22	11.25 ± 4.77	13.17 ± 7.65
Degree (mild/severe)^a^	5/17	3/17	6/20
TNSS (12 h) baseline	5.50 ± 3.10	6.35 ± 3.36	4.65 ± 3.72
TNSS (2 weeks) baseline	7.50 ± 2.79	7.90 ± 3.16	7.12 ± 3.31
CSMS (12 h) baseline	3.07 ± 1.58	3.75 ± 2.17	2.81 ± 2.25
CSMS (2 weeks) baseline	4.09 ± 1.66	4.40 ± 1.69	3.58 ± 2.10
RQLQ baseline	2.46 ± 0.97	2.54 ± 1.03	2.11 ± 1.25
Daily diary (first 3 days)	3.18 ± 1.68	4.38 ± 1.48	3.52 ± 2.12

^a^Severity of allergy is determined by having symptoms more than 4 days/week and more than 4 weeks/year.

The study was registered as a clinical trial (DRKS00015804, registered on 2/11/2018). Patients provided written informed consent before participating in the study.

Inclusion criteria of our study were a medical record of allergic rhinitis and age between 18 and 60 years. Exclusion criteria were a medical history of diabetes, gastrointestinal diseases, use of antibiotic medication in the last 6 weeks, pregnancy, psychiatric or neurological history, perennial allergic rhinitis, chronic rhinosinusitis, or any other chronic nasal conditions such as anatomical alterations (e.g., septum deviation or perforation).

To determine the sample size, we considered previous studies on allergic symptoms (for details see study protocol^19^). Power calculations resulted in a total sample size of n = 80 (desired power of 0.80, alpha error probability of 0.05). To account for possible drop-outs, we calculated with a total sample size of 120 participants.

### Study design, interventions, and procedure

The study design consists of a randomized placebo-controlled four-arm study of the OLP-treatment and its effects on symptoms of allergic rhinitis. The OLP treatment was compared to a conventional double-blind placebo arm, a TAU control arm, and a double-blind drug condition (probiotic treatment, PROB) (see Fig. 1). The results for the probiotic treatment will be reported in a separate publication, which examines the question whether a probiotic treatment has an effect on symptoms in allergic rhinitis. The current study focused only on placebo effects and investigates the impact of placebos with and without concealment. However, the probiotic arm was necessary to establish a double-blind condition. The study was conducted at the Medical School Berlin (MSB), Berlin, Germany. The trial protocol of this study has been published^19^.

We used drops for both placebo conditions (OLP and double-blind placebo) that included the carrier solution of the probiotic treatment (lactose-monohydrate, glucose-monohydrate). The drops were indistinguishable in color, smell, and taste from the probiotic.

The study was announced as research about psychophysiological interactions during the treatment of seasonal allergic symptoms. Participants were informed that they were assigned to different groups to receive a probiotic treatment or a placebo in a double-blinded fashion, or OLP, or no treatment. The probiotic treatment was offered to all participants after the end of the trial.

All participants received the same information and instructions (no additional information, e.g., for OLP or PROB group, after group assignment). After signing informed consent patients first completed questionnaires to measure the allergic symptoms (see below). Subsequently all participants were told that placebos are inactive substances containing no medications, but that both deceptive as well as non-deceptive placebos may still be powerful. We further explained that a possible mechanism for placebo responses may be classical conditioning, that a positive attitude may be helpful, but is not necessary, and that taking placebos faithfully is important. This information was analogue to previous studies^5^. Furthermore, we explained what probiotics are, that they can also be found in certain food such as sauerkraut or yoghurt, and that they are known to have beneficial effects for health. Subsequently we randomized the participants into one of the four arms of the study by opening an opaque envelope. Randomization was based on a computer-generated random number sequence (independent researcher). In the first arm patients received the probiotic treatment for 4 weeks (PROB). In the second arm identically looking placebo drops (double blind placebo, DBP) were given to the participants (PROB and DBP conditions were kept secluded and only broken after full evaluation of the study). The third arm was the OLP condition, here the patients received the same placebo, but were told that these drops were a placebo. The last arm was the treatment-as-usual condition (TAU). In all conditions participants were instructed to continue their usual allergic pharmacological medication.

In the DBP and OLP condition participants were instructed to take 30 drops three times a day. OLP or DBP (or PROB) groups received no further instruction or information. All participants were asked to complete a daily diary about the burden of allergic symptoms.

After 4 weeks all participants were invited for a second visit with a blinded experimenter, in which participants completed again questionnaires with respect to the allergic burden.

### Primary endpoints

The primary outcome measure was the Combined Symptoms and Medication Score (CSMS)^20,21^, which is recommended by the European Academy of Allergy and Clinical Immunology (EAACI)^20,22^. The CSMS assesses allergic symptoms including nose and eyes symptoms with respect to the last 12 h and the last two weeks. Symptoms are rated ranging from “0” (= no symptoms) to “+++” (= strong symptoms) (0 to 3). Thus, lower scores represent reduced symptom burden. It also asks the use of medication, but because our patients are instructed to continue with their regular medication, we computed the CSMS without medication scores.

A further primary endpoint measure was quality of life measured with the Rhinitis Quality of Life Questionnaire (RQLQ). This instrument includes 28 questions within seven fields to assess health-related quality of life in allergic rhinitis (activity limitations, sleep impairment, non-nasal/eye symptoms, practical problems, nasal symptoms, eye symptoms, and emotional problems). Participants have to indicate their responses on Likert scales from “0” (= symptoms did not bother me) to “6” (= symptoms bothered me extremely). Lower scores indicate improved health related quality of life. The RQLQ is widely used and has been validated before^23^.

### Secondary endpoints

A secondary endpoint measure is the Total Nasal Symptoms Score (TNSS). The TNSS is well-known to describe symptoms in allergic rhinitis. In contrast to other questionnaires the TNSS focuses on nasal symptoms. Participants have to rate five different nasal symptoms (e.g., itching or sneezing) on a scale from “0” (= not at all) to “3” (= symptoms difficult to bear, interfere with activities) with respect to the last 12 h and the last two weeks. Hence, lower scores reflect less symptoms. The TNSS is well-known and has been validated for many languages^24^.

In addition, we asked the participants to complete a daily diary. Patients had to use visual-analogue scales (VAS) to measure the daily burden of allergic symptoms^25^ (general feeling with respect to their allergic symptoms). The VAS consisted of a 10 cm long straight line with the ends “very good” to “very bad” (low scores indicate less symptoms).

### Statistical analyses

To test the effects of our treatment we applied generalized estimating equations (GEE) for repeated measures. The GEE includes an autoregressive correlation structure that considers correlation between repeated measurements on the same participant. Furthermore, to ensure that the groups are comparable at the beginning of the study, we tested whether baseline scores between the groups were different (Wilcoxon test). We also tested differences between symptoms before and during the pandemic (Wilcoxon test). Diary data was analyzed by comparing the mean of the first three days with the mean of the last three days.

### Ethical approval

The study adhered to the Declaration of Helsinki and was approved by an ethics committee. All participants gave written informed consent.

## Results

### Effects of OLP on allergic symptoms

**Figure 2:**
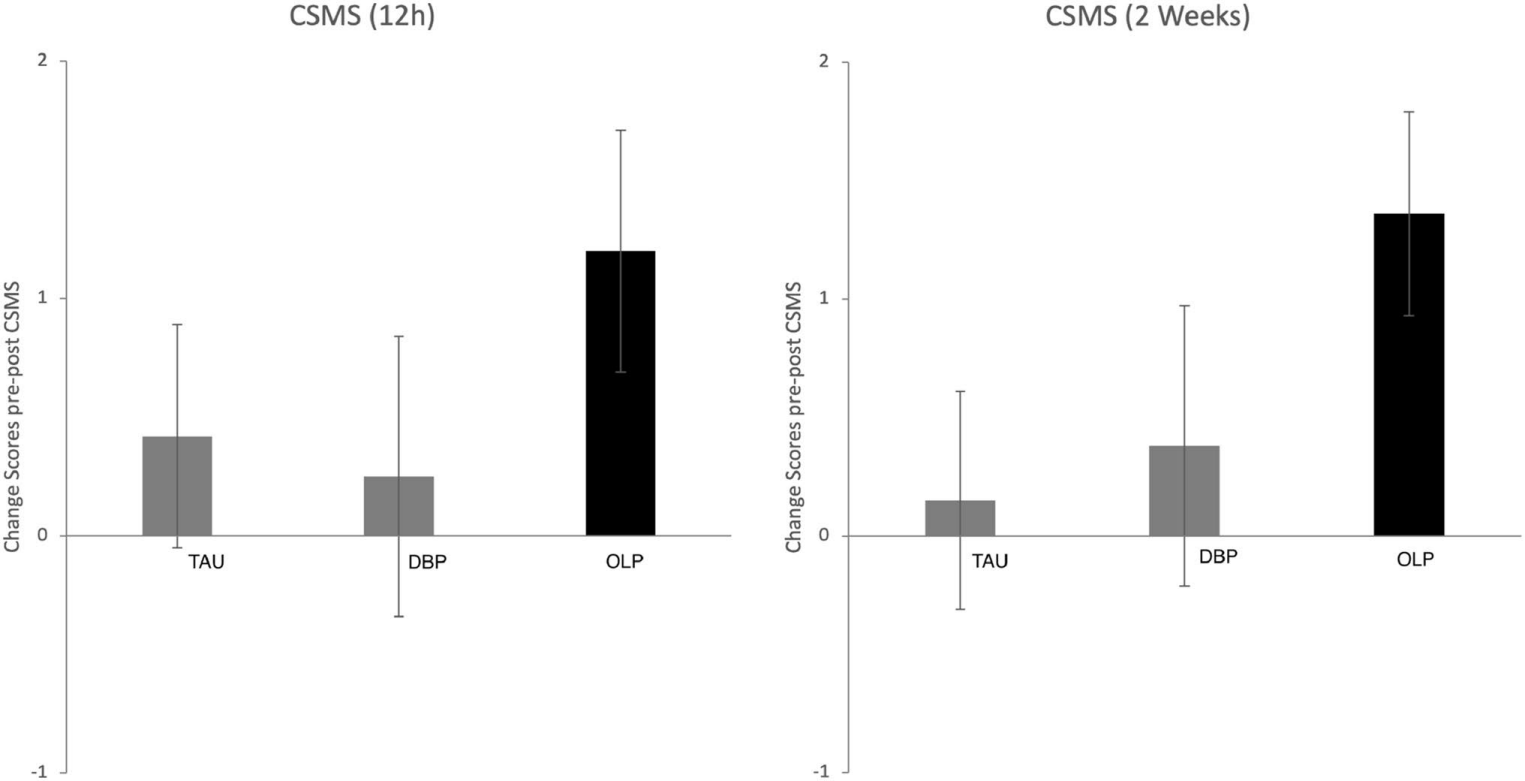
Outcomes at 4 weeks endpoint for primary outcome measure CSMS (change scores from pre to post, bars represent standard errors).

Baseline data revealed no differences with respect to the different arms (see Table 1). Analyzing the CSMS scores from pre to post showed greater improvements for the OLP group compared to the TAU and DBP groups when participants rated their symptoms over the past 12 h (change scores OLP: 1.20 ± 2.41, DBP: 0.25 ± 2.65, TAU: 0.42 ± 2.39; GEE model: Wald x^2^ = 14.32, p = 0.014, Φ = 0.46; see Fig. 2). Similar results were shown when participants had to evaluate the last 2 weeks (GEE: Wald x^2^ = 17.13, *p* = 0.004, Φ = 0.50; see Table 2). Results for the RQLQ also showed stronger improvements for OLP (OLP: 0.77 ± 1.09, DBP: 0.41 ± 1.36, TAU: 0.28 ± 0.95; GEE: Wald x^2^ = 18.62, *p* = 0.002, Φ = 0.52).

**Table 2:** Outcome at 4 weeks endpoint (mean ± standard deviations of change scores).

	Open-label placebo	Double-blind placebo	Treatment-as-usual
TNSS (12 h)	2.41 ± 3.81	− 0.40 ± 4.52	− 0.19 ± 3.95
TNSS (2 weeks)	1.18 ± 3.50	0.25 ± 4.70	0.42 ± 3.65
CSMS (12 h)	1.20 ± 2.41	0.25 ± 2.65	0.42 ± 2.39
CSMS (2 weeks)	1.36 ± 2.03	0.38 ± 2.65	0.15 ± 2.32
RQLQ	0.77 ± 1.09	0.41 ± 1.36	0.28 ± 0.95
Daily diary (first/last 3 days)	− 0.11 ± 3.25	0.78 ± 1.93	0.45 ± 2.62

**Figure 3:**
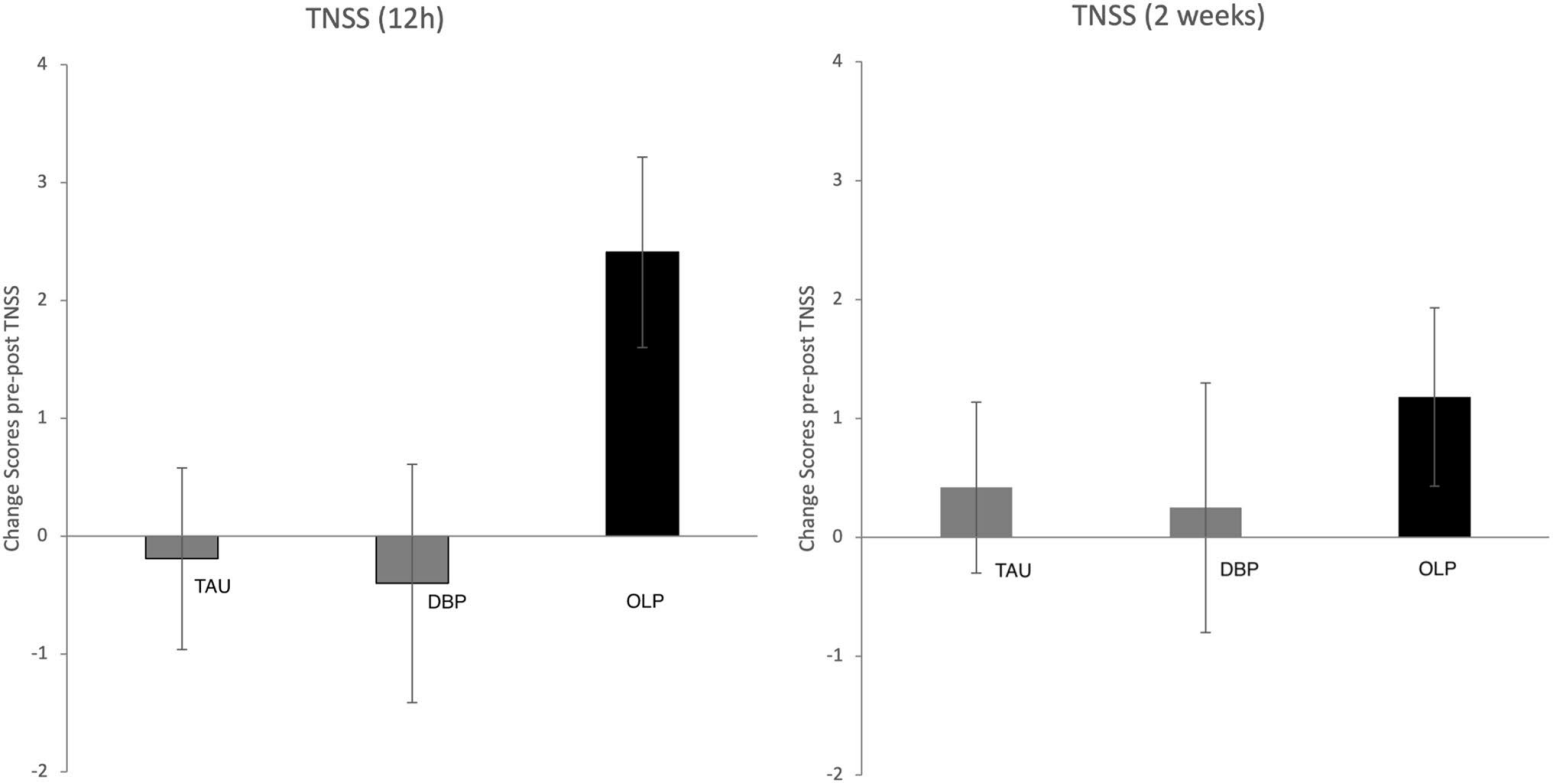
Outcomes at 4 weeks endpoint for secondary outcome measure TNSS (change scores from pre to post, bars represent standard errors).

TNSS results revealed improved scores for OLP relative to DBP and TAU (change scores for last 12 h; GEE: Wald x^2^ = 18.58, *p* = 0.002, Φ = 0.52; see Fig. 3). This is supported by the TNSS scores for the last 2 weeks but these results failed to reach the level of significance (GEE: Wald x^2^ = 5.06, *p* > 0.10, Φ = 0.27). Analysis of the diary data demonstrated an effect at borderline for OLP (GEE: Wald x^2^ = 11.08, *p* = 0.05, Φ = 0.40).

We further followed our a priori hypothesis and performed an analysis to test whether the OLP condition is superior to TAU (Wilcoxon test). Results revealed that OLP improved more than TAU for CSMS of the last two weeks with a medium effect size (Z = − 2.17, *p* = 0.030, r = 0.31), whereas the comparison for CSMS of the last 12 h failed to reach the level of significance (Z = − 1.40, *p* = 0.161, r = 0.20). OLP was also superior (with medium effect sizes) for outcome measure RQLQ (Z = − 2.07, *p* = 0.038, r = 0.30) and TNNS (12 h, Z = –2.86, *p* = 0.004, r = 0.41; 2 weeks, Z = − 0.95, *p* = 0.343, r = 0.14).

When comparing OLP with DBP we found that OLP was superior to DBP with respect to TNSS (12 h, Z = − 2.68, *p* = 0.007, Bonferroni corrected *p* = 0.021, r = 0.41; TNSS 2 weeks, *p* > 0.10) and showed a trend for significance for CSMS (12 h, Z = − 1.67, *p* = 0.095, corrected *p* > 0.10, r = 0.26; 2 weeks, Z = − 1.80, *p* = 0.073, corrected *p* > 0.10, r = 0.28) (RQLQ, *p* > 0.10). Comparisons between DBP and TAU revealed no significant differences (all *p* > 0.10). For the daily diary we found no significant effects (all *p* > 0.10).

### Effects of COVID-19 on allergic symptoms and treatments

Since a part of our sample was included before the pandemic onset in 2020, we were able to compare the allergic burden in general as well as the impact of placebo treatments before and during (or end of) the pandemic. Results demonstrated that patients showed significantly stronger allergic symptoms prior to the pandemic (for CSMS, TNSS, and RQLQ, considering all four arms of the study). Moreover, we found that the impact of the OLP treatment was also greater before the pandemic (see Tables 3 and 4). Because there were too few patients in the DBP group prior to the pandemic, we were unable to assess the impact of Covid-19 on the effects of a conventional placebo.

**Table 3:** Comparison of allergic symptoms before and after the COVID-19 pandemic (baseline data; bold data marks significant results, Wilcoxon tests, including all 4 arms of the study, N = 89).

	Before pandemic (N = 25)	During pandemic (N = 64)	
TNSS (12 h)	6.71 ± 4.23	5.02 ± 3.11	z = − 1.51, *p* = 0.130
TNSS (2 weeks)	8.57 ± 3.35	7.13 ± 2.86	**z = − 2.21, *p* = 0.027**
CSMS (12 h)	3.58 ± 2.19	3.02 ± 1.94	z = − 1.04, *p* = 0.299
CSMS (2 weeks)	4.66 ± 2.03	3.79 ± 1.78	**z = − 2.06, *p* = 0.039**
RQLQ	2.75 ± 1.10	2.18 ± 1.06	**z = − 2.44, *p* = 0.015**

**Table 4:** Comparison of OLP effects before and after the COVID-19 pandemic (change scores, bold data marks significant results, Wilcoxon tests, N = 22 (OLP group only)).

	Before pandemic (N = 6)	During pandemic (N = 16)	
TNSS (12 h)	4.83 ± 2.48	1.50 ± 3.88	**z = − 2.04, *p* = 0.041**
TNSS (2 weeks)	2.83 ± 3.43	0.56 ± 3.42	z = − 1.26, *p* = 0.207
CSMS (12 h)	2.50 ± 1.87	0.84 ± 1.92	z = − 1.45, *p* = 0.147
CSMS (2 weeks)	2.75 ± 1.75	0.40 ± 1.03	z = − 0.45, *p* = 0.651
RQLQ	1.75 ± 0.44	0.40 ± 1.03	**z = − 2.84, *p* = 0.004**

## Discussion

The present study aimed to test the efficacy of OLPs to relieve symptoms of allergic rhinitis. Our results showed that OLP improved symptoms more than DBP and TAU conditions.

OLP effects have been reported with respect to different clinical and non-clinical symptoms, but usually the impact of OLPs is compared with a TAU. This study is trying to compare the effects of an OLP treatment not only with TAU, but also with a double-blinded placebo condition. Our results replicated previous findings of an OLP treatment on symptoms in allergic rhinitis compared with TAU^9,10^. In contrast to those previous studies, we here show beneficial effects of OLPs based on the CSMS questionnaire, which is recommended to test for changes in allergic symptoms, as well as the widely used TNSS, which allows better comparability with other studies. Furthermore, the OLP group improved for health-related quality of life (RQLQ). However, for nasal symptoms measured with the TNSS we did not find significant effects when asking to assess the time of the last 2 weeks. This might be explained by difficulties in assessing the allergic burden of the past.

Remarkably, we found that OLP effects were superior to conventionally concealed placebos, which showed no effects when compared with TAU. Thus, it is surprising that we did not see any “classic” placebo effects here. Considering that waxing and waning are characteristic for allergic symptoms, placebo effects in allergic rhinitis are well-known^16–18^. A recent metanalysis revealed that in seasonal allergic rhinitis placebo responses resulted in improvements of about 15%, whereas active treatments such as antihistamines or corticosteroids showed 1.56 and 2.71-fold greater improvement than placebo^18,26^. One could speculate that the absence of the conventional placebo effect here may be accounted to the dosage form. Possibly other kinds of placebos (e.g., pills) might have induced a placebo effect. Another reason may point to group differences with respect to positive expectations. Although all groups received identical instructions, expectations may still be different. The OLP group received bottles with the label “Placebo”, whereas the bottles of the DBP group had “Placebo or Probiotic” labels. In this DBP condition participants (who took the drops for a relatively long time) may have thought that they received the treatment rather than a placebo. This view may also be supported by the non-sweet taste of the drops. Therefore, the placebo instructions might not have any effects for those participants. Future studies should ask participants at the end of the study what they think they actually had received.

The present study is one of the first 4-arm studies comparing the effects of open-label with concealed placebos. Locher et al. compared OLPs with conventionally concealed placebos and a no treatment control on pain tolerance. Their results showed that OLPs and placebos with deception did not differ in subjective heat pain intensity or unpleasantness ratings^27^. Lembo et al. compared the effect of OLPs with TAU and conventionally concealed placebos on patients with irritable bowel syndrome. They found that OLP was superior to TAU in improving symptoms and similar to the effect of placebos with deception^28^. Our results supplement these results by demonstrating that OLP was even better than DBP. The results suggest that the way OLPs work may be different compared with the mechanisms of DBP. For example, expectancy is often used to explain the conventional placebo effect, but recent research suggests that this mechanism may not account for OLP effects (e.g.,^28,29^). Although the exact mechanisms of OLP effects remain to be cleared, one could speculate that other mechanisms seem to be more important for OLPs and that these mechanisms may distinguish OLP from DBP treatments^30^. However, based on the present data we feel unable to address the mechanisms of OLPs more in detail.

While we could replicate OLP effects on symptoms in seasonal allergic rhinitis, a recent study did not find any effects of OLPs for those patients^31^. Several differences might account for this lack of effects. First, the study by Kube et al. provided OLP remotely. Thus, personal interaction was very reduced by using online ways to communicate and perform the measurements. A second point may be even more important. Kube et al. conducted their study during the Covid-19 pandemic. This may be very influential.

In which ways may the Covid-19 pandemic have affected the results? The present study included patients both before and during the end of the pandemic, allowing us to compare general allergic symptoms (our baseline data) and also the efficacy of OLP effects before and during the pandemic. The results demonstrate that allergic symptoms in general were reduced during the Covid-19 pandemic, which is in line with recent studies^32–34^. A reason for this improvement of symptoms may be the reduced confrontation with allergic pollen because of lock-down situations or due to the obligation of wearing a mask in public. In addition, psychological factors may explain this finding, since nose related symptoms of allergies are similar to Covid-19 symptoms and individuals may have neglected these symptoms because they did not want to think about the chance to be infected by the virus.

Not only were general allergic symptoms affected, but we also found that the OLP effects were weaker during the Covid-19 pandemic. Although we have to consider that this comparison is based on relatively few participants, one could speculate why individuals during the Covid-19 pandemic might be less prone to OLP effects. For example, psychological reasons might explain these differences (see above). Furthermore, the smaller OLP effects may be explained by statistical effects. The reduced baseline allergic symptoms during the Covid-19 pandemic may have given less opportunities to show improvements. Unfortunately, we did not have sufficient data to assess the impact of the Covid-19 pandemic on concealed placebos.

While we observed significant effects of OLPs on allergic symptoms using TNSS and CSMS, OLPs showed only weak effects on the diary data. One explanation might be that the diary data ask for VAS scores of the situation in a very general way (in contrast to TNSS and CSMS, which ask for specific symptoms). A previous study found effects also on diary data when asking more specifically about allergic symptoms^10^. Interestingly, a recent study of OLP effects on allergy symptoms observed more pronounced effects when participants were asked to rate the frequency rather than the severity of symptoms^12^. In the current study we similarly found the highest effect sizes for the TNSS, which does not ask about symptom severity as directly as the CSMS does. Remarkably, we found the weakest results for the diary data that asked for the general feeling with respect of the allergic, which may be interpreted as rating the severity of symptoms. These differences may point to the importance of the way how to assess self-report data in OLP studies.

Some limitations of this study have to be mentioned. The data of the current study relies on self-report questionnaires. Given that OLP treatments cannot be administered in a double blinded way, response bias might be a problem. Although we tried to address this issue by adding a conventional placebo condition, further studies are needed to assess the impact of OLPs. In particular, studies using physiological data sources could help to test the impact of OLPs on allergic symptoms. For example, Leibowitz et al. examined physiological allergic reactions in an histamine skin prick test and found an effect for OLP in participants with a strong belief in placebos^11^. Furthermore, future studies are needed to replicate this finding, using, for example, increased sample sizes.

The present study aimed to test the efficacy of OLP in patients with seasonal allergic rhinitis. Our results demonstrated that OLPs improved self-reported allergic symptoms in a 4-week trial, whereas a conventional placebo failed to show any effects. The results point to different mechanisms of placebo treatments. We conclude that the results encourage further research on the effects of OLPs.

## Data Availability

The data presented in this study are available on request from the corresponding author.
